# Mechanical property degradation of X80 pipeline steel due to microbiologically influenced corrosion caused by *Desulfovibrio vulgaris*

**DOI:** 10.3389/fbioe.2022.1028462

**Published:** 2022-11-07

**Authors:** Zhong Li, Jike Yang, Huihua Guo, Sith Kumseranee, Suchada Punpruk, Magdy E. Mohamed, Mazen A. Saleh, Tingyue Gu

**Affiliations:** ^1^ Department of Chemical and Biomolecular Engineering, Institute for Corrosion and Multiphase Technology, Ohio University, Athens, OH, United States; ^2^ Corrosion and Protection Center, University of Science and Technology Beijing, Beijing, China; ^3^ PTT Exploration and Production, Bangkok, Thailand; ^4^ Research and Development Center, Saudi Arabian Oil Company, Dhahran, Saudi Arabia

**Keywords:** MIC, mechanical property, sulfate reducing bacteria, tensile test, H_2_S

## Abstract

Apart from pinhole leaks, MIC (microbiologically influenced corrosion) can also cause catastrophic failures such as pipe ruptures and support beam collapses due to mechanical property degradation or stress corrosion cracking. In this work, X80 pipeline steel dogbone coupons and square coupons were immersed in 150 ml broths containing *Desulfovibrio vulgaris*, a common corrosive sulfate reducing bacterium (SRB), for up to 14 days. The headspace volumes in the anaerobic bottles were increased from 150 ml to 200 ml and 300 ml to increase MIC severity. After 14 days of SRB incubation in ATCC 1249 culture medium with X80 coupons at 37°C, the sessile cell counts were 6.5 × 10^7^ cells cm^−2^ for 150 ml, 2.3 × 10^8^ cells cm^−2^ for 200 ml and 1.4 × 10^9^ cells cm^−2^ for 300 ml headspace volumes, respectively owing to reduced H_2_S cytotoxicity in the broth with a larger headspace because it allowed more biogenic H_2_S to escape from the broth. Weight losses were 1.7 mg cm^−2^, 1.9 mg cm^−2^ and 2.3 mg cm^−2^ for 150 ml, 200 ml and 300 ml headspace volumes, respectively. The corresponding pit depths were 2.6 μm, 4.2 μm and 6.2 μm for 150 ml, 200 ml and 300 ml headspace volumes, respectively. Electrochemical impedance spectroscopy (EIS), linear polarization resistance (LPR) and potentiodynamic polarization results corroborated the increasing weight loss and pitting data trends as a result of increased headspace. Tensile testing of dogbone coupons after the 14-day SRB immersion test indicated that more severe MIC pitting led to a higher ultimate strain loss by up to 23% (300 ml headspace) compared to the abiotic control, while the ultimate strength losses for all headspace volumes were quite small (3% and lower).

## Introduction

There is growing awareness of MIC (microbiologically influenced corrosion) threat to various assets in marine, oil and gas, and water utilities industries, *etc*. It is widely believed that MIC accounts for at least 20% of all of corrosion losses (Jia et al., 2017; Xu et al., 2017; Jia et al., 2019a). NACE International estimated that the total cost of corrosion is around US$2.5 trillion/year globally, and MIC accounts for 20%–40% (Beavers and Thompson, 2006; Wolodko et al., 2018; Salgar-Chaparro et al., 2020). The Aliso Canyon gas leak between 2015 and 2016 caused a major environmental disaster with a massive emission of methane gas that is a very potent greenhouse gas. The leak was attributed to metal well casing failure due to soil MIC (CPUC and DOGGR, 2019). In addition to pinhole leaks which was likely the case for the 2006 Alaska Pipeline leak (Jacobson, 2007), MIC can cause mechanical property degradation, leading to metal fracturing/rupturing/collapsing and cracking that reduce equipment service lifespan (Enning and Garrelfs, 2014; Sherman et al., 2015). Most MIC studies so far focused on pitting corrosion. There is a lack of studies on the impact of MIC on mechanical property degradation. In practical applications, disastrous consequences such as pipeline ruptures and support beam collapses can be caused by mechanical property degradation with MIC as the root cause.

SRB (sulfate reducing bacteria) are a major type of microbes that cause MIC. SRB can acquire energy by oxidizing organic substances or H_2_ for reducing sulfate (SO_4_^2−^) to hydrogen sulfide (H_2_S) and other sulfide species (Lovley and Phillips, 1994; Promnuan and Sompong, 2017). When sulfate acts as the electron acceptor and lactate (soluble organic carbon) as the electron donor for SRB respiration, the redox reaction occurs entirely inside SRB cells to generate energy (Jacobson, 2007; Xu and Gu, 2014; Li et al., 2018).

$${CH_{3}\;CHOHCOO}^{-}{+H}_{2}O\to {CH_{3}\text{COO}}^{-}{+CO}_{2}{+4H}^{+}{+4e}^{-}\;\left({E{}^{\circ}}^{\prime }=-430\;\text{mV}\right)$$ (1)

$${\text{SO}}_{4}^{2-}{+9H}^{+}{+8e}^{-}\to {\text{HS}}^{-}{+4H}_{2}O\;\left({E{}^{\circ}}^{\prime }=-217\;\text{mV}\right)$$ (2)

In the two half-reactions above, E°' is the reduction potential vs. SHE (standard hydrogen electrode) at 25°C, pH 7, and 1 M solutes (or 1 bar gases) (Thauer et al., 2007). The actual respiration of sulfate using lactate as electron donor is more complicated. It usually involves lactate oxidation to produce pyruvate, and then pyruvate oxidation to yield H_2_ with concomitant ATP (adenosine 5′-triphosphate) production. H_2_ serves as electron donor for sulfate reduction (Peck, 1993; Smith et al., 2019; Zhou et al., 2022).

SRB sessile cells require energy to maintain themselves even when they are not growing. When there is a lack of carbon source in the local environment near the bottom of an SRB biofilm, elemental iron can provide electrons for SRB survival, which leads to MIC. *E*°′ of Fe^2+^/Fe is similar to that of acetate + CO_2_/lactate (Thauer et al., 2007). This means elemental Fe is as energetic as lactate.

$$4\text{Fe}\to {\text{Fe}}^{2+}{+8e}^{-}\;{(E}^{o^{\prime }}=-447\;\text{mV})$$ (3)

$${\text{SO}}_{4}^{2-}{+9H}^{+}{+8e}^{-}\to {\text{HS}}^{-}{+4H}_{2}O\;\left(E^{o^{\prime }}=-217\;\text{mV}\right)$$ (4)

The cell potential (Δ*E* ^o^′) of the redox reaction combining Reactions (3) and (4) above is +230 mV, which results in a negative Gibbs free energy change, indicating that the overall corrosion reaction is thermodynamically favored (Gu et al., 2015). Electrons from extracellular iron (insoluble) oxidation must be transported across the SRB cell membrane to the SRB cytoplasm for sulfate reduction (Eaktasang et al., 2016; Lv and Du, 2018). This kind of cross-cell membrane electron transfer process is known as extracellular electron transfer (EET), an important topic in microbial metabolism for energy production (Lovley and Phillips, 1994; Li et al., 2018; Gu et al., 2021; Huang et al., 2022). *D*. *vulgaris* MIC of carbon steel observes the EET-MIC theory according to the evidence provided by carbon source starvation tests and electron mediator tests in the literature (Xu and Gu, 2014; Dou et al., 2019; Wang et al., 2020). 2H^+^/H_2_ is used as an electron shuttle (i.e., H_2_ cycling) for hydrogenase-positive SRB such as *D. vulgaris* to donate electrons for sulfate reduction (Peck, 1993). 2H^+^/H_2_ electron shuttle can bridge Reactions (3) and (4) with H_2_ cycling, which is consistent with the cathodic depolarization theory (Li et al., 2018).

Experimental data have rather conclusively shown that H_2_S is not the cause of *D. vulgaris* (a typical SRB strain) corrosion of carbon steel at circumneutral broth pH (Wang et al., 2020). Typical SRB MIC of carbon steel tests are not like abiotic H_2_S corrosion which involves acidic pH with a large amount of H_2_ produced (Jia et al., 2019a).

In the past, most investigations focused on MIC pitting. Not many studies paid attention to MIC impact on the degradation of mechanical properties. MIC pitting of metal surfaces weaken the metals (Unsal et al., 2016; Jia et al., 2019a; Jia et al., 2019b; Wang et al., 2020). Pit density and pit depth both impacted the mechanical properties of materials (Tang et al., 2014; Sheng and Xia, 2017; Javed et al., 2020a; Javed et al., 2020b; Zhang et al., 2020). In abiotic corrosion studies, it was found that corrosion activity degraded the ultimate strength of steel (Saad-Eldeen et al., 2012). It is suggested that when SRB are present, some engineering materials are likely to fail in a relatively shorter time than in an abiotic environment (Javaherdashti, 2011). It has been reported that the ultimate strength and ultimate strain were reduced significantly in the presence of the *Pseudomonas* species due to the biofilm formation and the resultant MIC process (Vaidya et al., 1997; Huang et al., 2022). In another study, the presence of corrosive *Bacillus megaterium* bacterium decreased the mechanical properties such as yield stress, ultimate strength and elongation of an Al-Cu alloy (Yousaf et al., 2015). Recently, it was found that moderately starved *D. vulgaris* biofilm degraded ultimate tensile strength and ultimate tensile strain of X80 carbon steel more than those with the biofilm without carbon source starvation because starvation made SRB sessile cells more eager to harvest electrons from Fe(0) *via* EET (Li et al., 2022).

It has been known that in carbon steel MIC by SRB, a larger headspace allows more H_2_S to escape from the broth. This reduces the H_2_S cytotoxicity in the broth, allowing better planktonic and sessile SRB growth, and thus leading to more severe MIC (Jia et al., 2019a). X80 carbon steel is widely used in many industries because of its low cost and ease of fabrication (López-Celvera et al., 2018; Zhang et al., 2019; Pereira et al., 2021; Tian and Pan, 2021). However, X80 steel pipelines may suffer from both MIC and mechanical property degradation caused by MIC (Alamri, 2020; Liu et al., 2022; Fu et al., 2022; Wasim and Djukic, 2022). This study investigated the effects of SRB sessile cell growth on MIC and the subsequent mechanical property degradation of X80 pipeline steel. In this study, dogbone coupons made of X80 carbon steel were used to investigate mechanical property degradation as a consequence of exposure to varied severity of MIC pitting by SRB, which was achieved by varying the headspace. After SRB exposure in anaerobic bottles, X80 dogbones were analyzed for MIC pitting and then tested on a tensile machine to measure mechanical property damages. Square X80 coupons were used to obtain weight loss. Square X80 coupons were also used as working electrodes in electrochemical glass cells to measure MIC severity electrochemically to corroborate weight loss and pit depth data trends from anaerobic bottles and to provide transient corrosion behavior.

## Experimental

### Preparation of X80 dogbone coupons and square coupons

**FIGURE 1:**
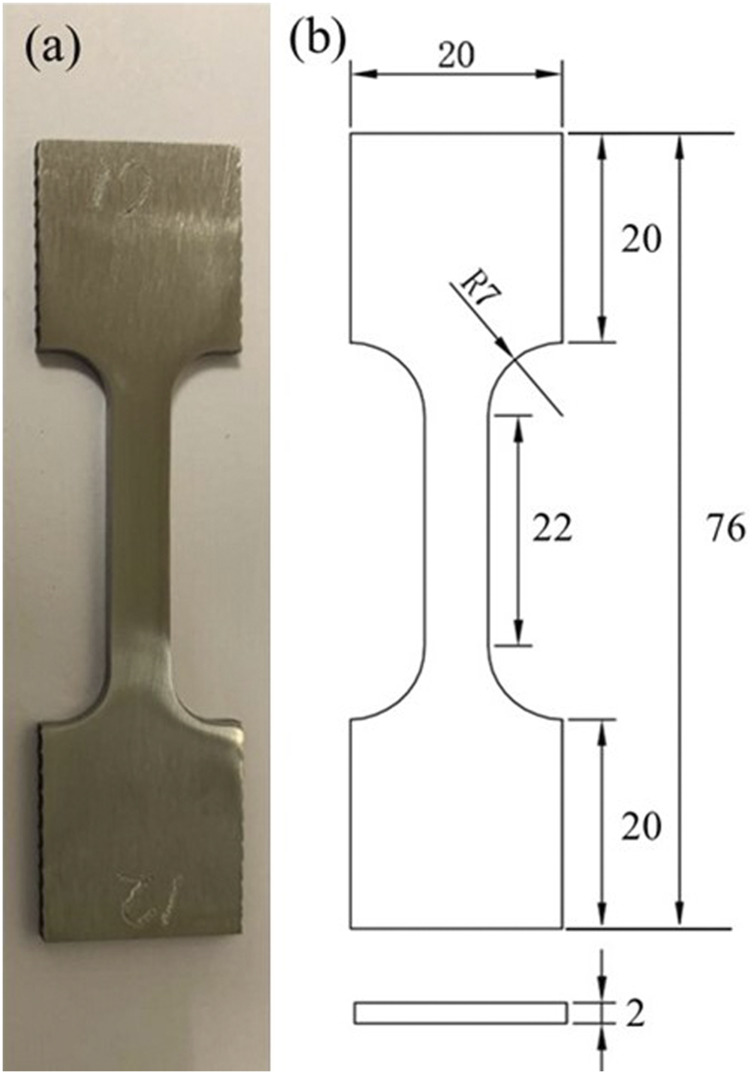
**(A)** Image of dogbone coupon of X80 carbon steel, and **(B)** its dimensions in mm.

The X80 steel composition is listed in Table 1. Dogbone coupons were too heavy to measure milligram weight loss accurately in this work. Thus, three square coupons, each with a 1 cm^2^ unpainted top surface (all other surfaces were covered with a polytetrafluoroethylene paint), were incubated without shaking in each anaerobic bottle with 150 ml SRB broth to obtain one MIC weight loss data point. Square coupons (1 cm^2^ exposed surface) were also used as working electrodes in electrochemical glass cells. Dogbone specimens were used to test the mechanical properties. The dimensions of the dogbone coupons (Figure 1) were based on the ASTM E8/E8M standard (ASTM-E8/E8M-13a, 2013). The dogbone coupons were polished to 1,200 grit by the supplier. Each dogbone coupon was painted with polytetrafluoroethylene, except for a middle section with a width of 6 mm and length of 22 mm which was exposed to the SRB broth on all four sides. The top surfaces of all the square coupons (including the abiotic control and electrode coupons) were sequentially polished with 180, 400 and 600 grit abrasive papers. After that, all the coupons were cleaned with pure isopropanol and dried under UV light for 20 min.

**TABLE 1:** Elemental composition of X80 steel (mass%).

C	Mn	Ni	Cu	Si	Mo	Cr	Nb	Ti	Fe
0.050	1.850	0.285	0.246	0.228	0.307	0.016	0.065	0.013	Balance

### Culture medium and inoculum

*D. vulgaris* (ATCC 7757), a common SRB strain in MIC research, was selected for this research. The culture medium was ATCC 1249 medium, which is a modified Baar’s medium for sulfate reducers. The culture medium pH was adjusted to pH 7 using a NaOH solution. The culture medium was sterilized in an autoclave at 121°C. After autoclaving, the culture medium was deoxygenated using filter-sterilized N_2_ sparging for more than 45 min. One hundred ppm (final concentration) L-cysteine was then added to the culture medium as an oxygen scavenger to reduce dissolved oxygen further and to mitigate possible slow oxygen leakage (Dou et al., 2019). Each anaerobic bottle was inoculated with 2 ml 3-day old SRB seed culture (grown in ATCC 1249 culture medium) before incubation at 37°C without shaking.

### Biofilm morphology and coupon weight loss

In this research, the X80 dogbone coupons were too large for SEM (scanning electron microscopy) imaging work and for obtaining accurate weight loss data. Thus, small X80 square coupons (1 cm^2^ exposed top surface) were used to obtain the biofilm SEM images and weight loss data after 14 days of incubation 37°C. Each 450 ml anaerobic bottle contained 150 ml deoxygenated culture medium (fixed) with either 150 ml, 200 ml or 300 ml headspace (adjusted using inert glass beads or Epoxy blocks).

A SEM machine (FEI Quanta 250, Hillsboro, OR, United States) was used to observe the biofilm morphology on square coupons. Before the SEM observations, the cells and corrosion products/biomass were gentled rinsed with a PBS (phosphate buffered saline) solution for 15 s each for 3 times before sessile cell counting, and then soaked in 2.5% (w/w) glutaraldehyde biocide solution for 8 h at 10°C to immobilize the biofilm on each coupon. Then, the coupons were sequentially dehydrated with 50% (v/v), 70%, 80% 90%, and 95% ethanol sequentially for 10 min at each concentration and finally with 100% ethanol for 0.5 h. Subsequently, the coupon surfaces were sputter coated with Au to provide electric conductivity before the SEM observations.

After the 14-day incubation, the square coupons for weight loss data were cleaned with a fresh Clarke’s solution to remove biofilms and corrosion products before weighing. Each weight loss data point was the average of 3 replicate coupons from the same anaerobic bottle.

### Electrochemical measurements

A potentiostat (Model VersaSTAT 3, Princeton Applied Research, Oak Ridge, TN, United States) was used to measure the electrochemical responses of the X80 working electrode (1 cm^2^ surface) in SRB broth. Each glass cell contained 150 ml deoxygenated culture medium (fixed) with either 150 ml, 200 ml or 300 ml headspace (adjusted using Epoxy resin as space filler). Each bottle was inoculated with 2 ml 3-day old SRB seed culture for static incubation at 37°C. A saturated calomel electrode (SCE) was used as the reference electrode, and a thin platinum plate (10 mm × 10 mm × 1 mm) was used as the counter electrode. The abiotic control glass cell had 150 ml culture medium and 300 ml headspace without SRB inoculation. There was no need to vary the headspace for the abiotic control because there was no biogenic H_2_S escape to the headspace.

Open circuit potential (OCP), LPR, EIS and potentiodynamic polarization analyses were performed. LPR was scanned at a rate of 0.1667 mV s^−1^ in the range of −10 mV to +10 mV vs. OCP. EIS was performed at OCP by applying a sinusoidal signal of 10 mV (amplitude) in the frequency ranging from 10^4^ to 10^–2^ Hz. Potentiodynamic polarization curves were measured at the end of the 14-day incubation from OCP to OCP −200 mV using one working electrode, and from OCP to OCP +200 mV using another working electrode in a replicate glass cell at a rate of 0.1667 mV s^−1^. The corrosion potential (*E*_corr_), corrosion current density (*i*_corr_), and anodic and (absolute) cathodic Tafel slopes (*β*_a_ and *β*_c_) were determined from a Tafel analysis of the polarization curves.

### Headspace gas measurements, sessile cell counts, pit depths, and tensile testing

Dogbone coupons were immersed in anaerobic bottles with 150 ml culture medium and varied headspace volumes (150 ml, 200 ml and 300 ml), and each anaerobic bottle contained one dogbone coupon. The headspace variation was achieved using Epoxy resin as space filler because the bottle volume and liquid culture medium volume were the same, but the headspace volume varied.

The concentration of H_2_S and total pressure in different anaerobic bottles were measured using a portable H_2_S sensor (GAXT-H-DL, BW Technologies, Calgary, Alberta, Canada), a digital manometer (Xplorer GLX-PS-2002, PASCO scientific, Roseville, CA, United States), respectively. The H_2_S sensor had an upper limit of 100 ppm (v/v). If a headspace sample had a higher concentration, dilution was required (Wang et al., 2020). A 125 ml anaerobic vial sealed with 1 atm air was injected with 10 ml headspace gas for 12.5X dilution. After mixing, a syringe was used to flush and flood the H_2_S sensor’s port with 40 ml of the headspace gas before taking a meter reading.

Sessile cells on a dogbone coupon were counted using a hemocytometer under a 400X microscope. Each dogbone coupon had a total area of 4 cm^2^ covered by the SRB biofilm. The biofilm biomass was first scrapped off the coupon and then suspended in a PBS buffer before counting. Because *D. vulgaris* cells were seen motile, they were easily distinguished from artifacts (Wang et al., 2020).

The biofilms and corrosion products on the dogbone coupon surfaces were removed using a fresh Clarke’s solution according to ASTM G1–03. After the removal, the maximum pit depth for each dogbone coupon was obtained under an InfiniteFocus Microscopy (IFM) machine (Model ALC13, Alicona Imaging GmbH, Graz, Austria).

After the pit depth analysis, tensile tests were performed on an electromechanical universal testing machine (E44.304, MTS system, MN, United States) on the same dogbone coupons.

## Results and discussion

### Surface and biofilm analyses using square coupons

**FIGURE 2:**
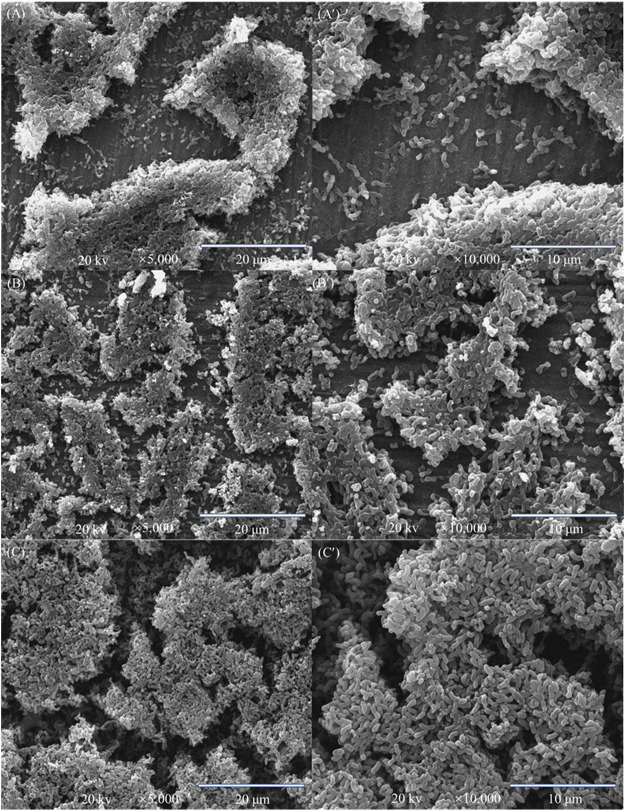
SEM biofilm images of X80 square coupon in 150 ml SRB broth with varied headspace volume after 14-day incubation: **(A,A′)** with 150 ml headspace at two magnifications, **(B,B′)** with 200 ml headspace, and **(C,C′)** with 300 ml headspace.

The SEM biofilm images in Figure 2 show the surface morphologies of the *D. vulgaris* biofilms for different headspace volumes after the 14-day incubation. The short rod shape is typical for *D. vulgaris,* consistent with SEM images in other studies (Unsal et al., 2022; Wang et al., 2022). Figure 2 indicates that the number of sessile cells increased with increasing headspace volume. This qualitative information is consistent with quantitative sessile cell count data on dogbone coupons discussed below. A larger headspace led to less H_2_S cytotoxicity in the broth and thus better SRB growth (Jia et al., 2019a).

### Weight losses using square coupons

**FIGURE 3:**
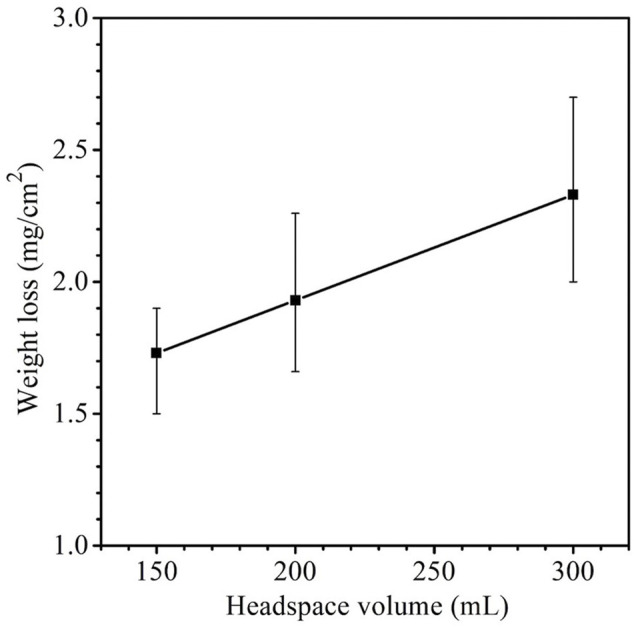
Weight losses of X80 in 150 ml SRB broth with varied headspace volume after 14-day incubation. (Each error bar represents standard deviation from 3 coupons in the same anaerobic bottle).

The weight losses for 150 ml, 200 ml and 300 headspace volumes were 1.7 ± 0.17 mg cm^−2^, 1.9 ± 0.33 mg cm^−2^ and 2.3 ± 0.37 mg cm^−2^, respectively (Figure 3). Although the neighboring weight loss data were close with fairly wide error bars as a result of the short-term test, the 150 ml and 300 ml weight losses had a *p*-value < 0.05, indicating that the weight increased with statistical significance when the headspace volume increased from 150 to 300 ml. These weight losses after SRB incubation were much larger than the 0.2 ± 0.05 mg cm^−2^ abiotic carbon steel weight loss obtained after 14 days of incubation in the deoxygenated ATCC 1249 culture medium without SRB inoculation. The increasing SRB MIC weight loss trend corresponds to the increasing sessile cell trend observed in Figure 2, which is consistent with EET-MIC, in which more sessile cells harvest more electrons from elemental iron, leading to more severe corrosion (Jia et al., 2018).

### Electrochemical tests using square coupons

**FIGURE 4:**
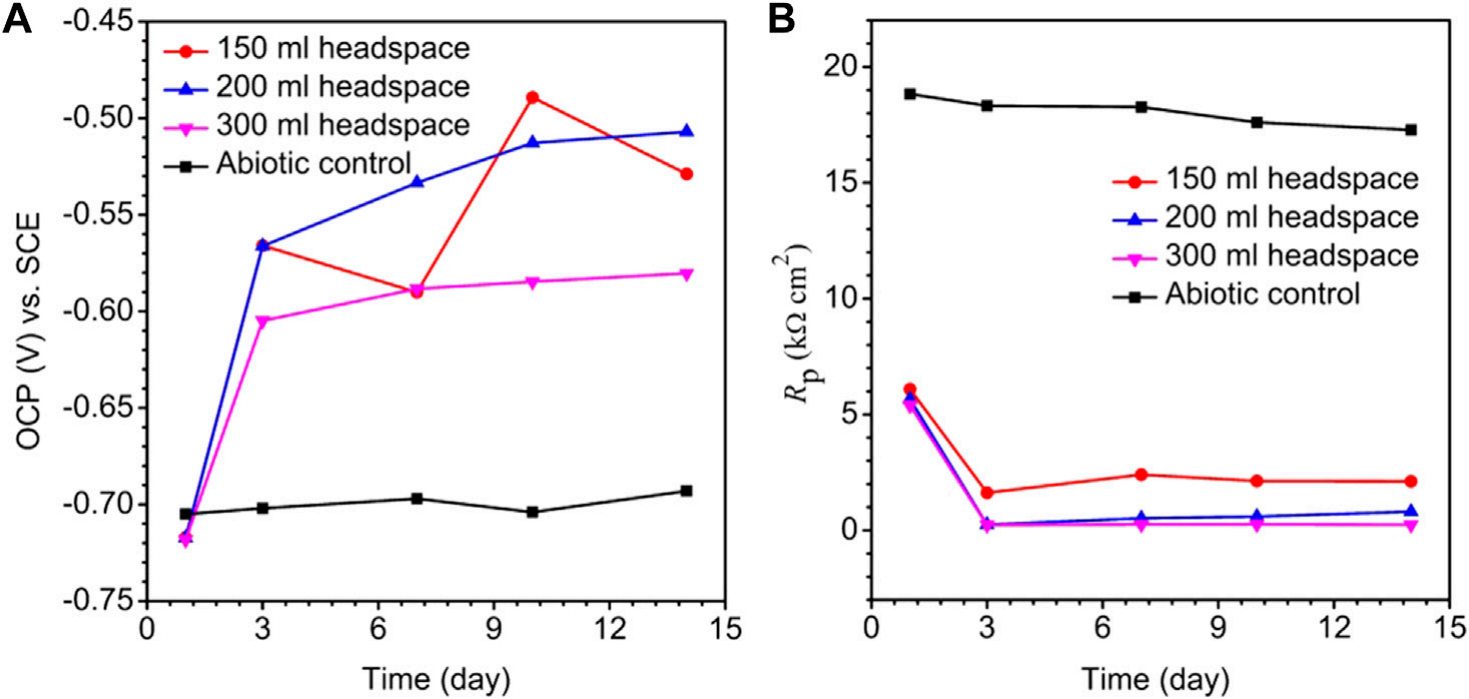
Variations of OCP vs. time **(A)** and *R*_p_ vs. time **(B)** for X80 in abiotic culture medium and in 150 ml SRB broth during 14-day incubation with headspace volumes of 150 ml, 200 ml and 300 ml (fixed 150 ml broth volume).

The OCP trends for different headspace volumes during the 14-day incubation of X80 electrode in the SRB culture medium are shown in Figure 4A. A lower OCP indicates a higher tendency for the working electrode to lose electrons. Figure 4A does not consistently indicate that a higher headspace volume had a lower OCP. This is not surprising for complicated SRB systems (Tran Thi Thuy et al., 2020). After all, OCP only indicates corrosion tendency, but the actual corrosion outcome relies on corrosion kinetics. The same observation was made previously in a study on biogenic H_2_S impact on carbon steel MIC by *D. vulgaris* in ATCC 1249 culture medium which included abiotic OCP (Jia et al., 2018).

Polarization resistance (*R*_p_) from LPR scans in Figure 4B describes the transient corrosion kinetics during the 14-day incubation. *R*_p_ is inversely proportional to corrosion rate (Cai et al., 2022). Figure 4B shows a large drop of *R*_p_ in the first 3 days, suggesting that as biofilm established on the metal surface, corrosion rate increased. The abiotic *R*_p_ curve for X80 in the deoxygenated ATCC 1249 culture medium remained around 17–18 kΩ cm^2^, much higher than the biotic *R*_p_ curves. Figure 4B also shows that *R*_p_ for the 300 ml headspace was the lowest, and *R*_p_ for 150 ml was the highest, indicating highest and lowest corrosion rate, respectively, which is consistent with weight loss data trend in Figure 3.

**FIGURE 5:**
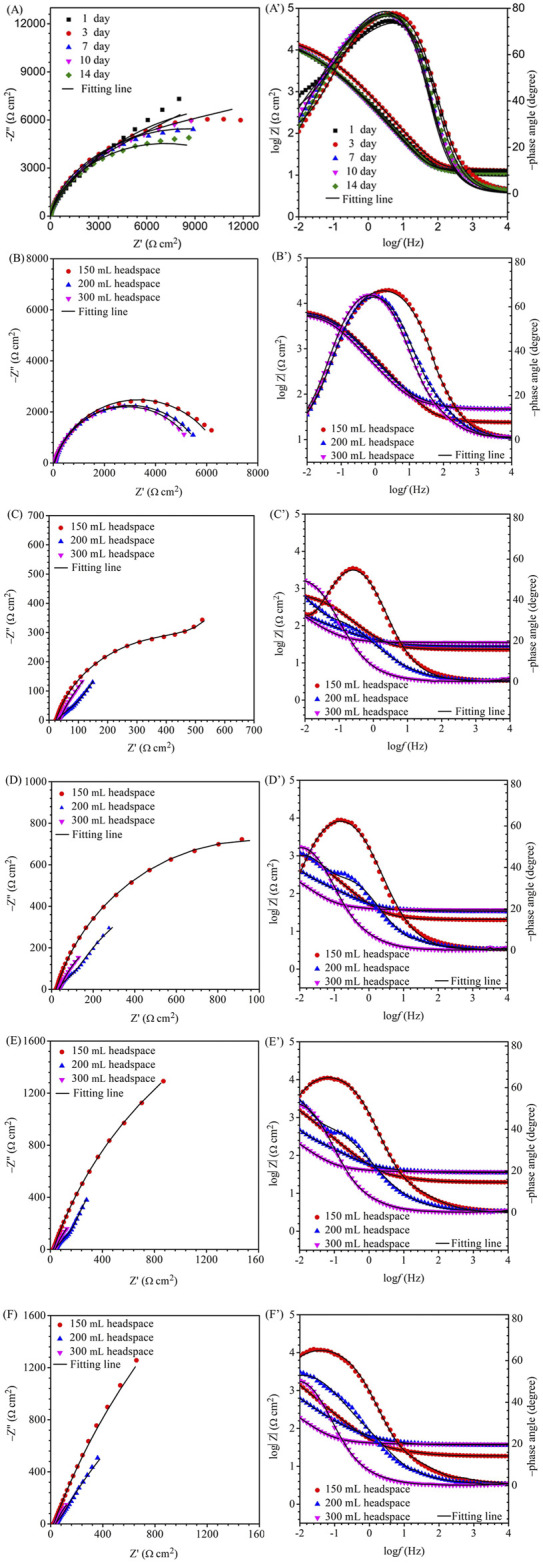
Nyquist and Bode plots for X80 in SRB broth during 14-day incubation with fixed 150 ml broth with varied headspace volume: **(A,A′)** abiotic control, **(B,B′)** first day biotic, **(C,C′)** third day biotic, **(D,D′)** seventh day biotic, **(E,E′)** 10th day biotic, and **(F,F′)** 14th day biotic EIS spectra.

**FIGURE 6:**
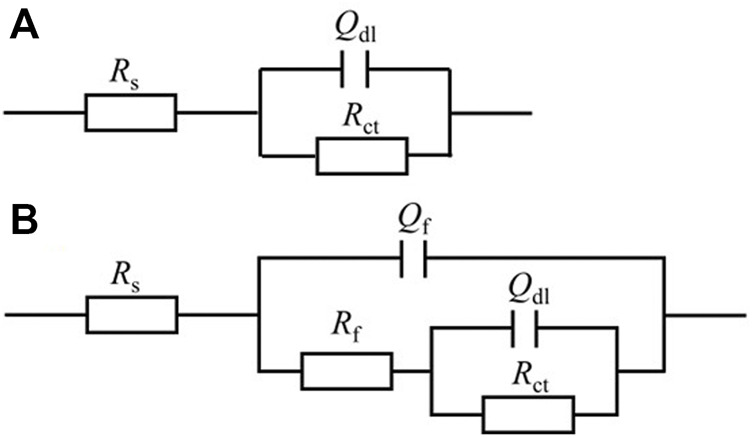
Equivalent electric circuits to model abiotic **(A)** and biotic **(B)** EIS spectra in Figure 5.

For EIS, the Nyquist and Bode plots of the abiotic and the biotic X80 coupons for different immersion times and different headspace volumes are shown in Figure 5. The abiotic EIS data in the deoxygenated ATCC 1249 culture medium were show the same trend with abiotic *R*_p_ trend. The Nyquist plots indicate a capacitive behavior. A larger diameter of the semi-circle in the Nyquist plot means a higher corrosion resistance in Figure 5A. The EIS data in Figure 5 were fitted with the equivalent electrical circuits in Figure 6. A simple one-time constant circuit was needed for the abiotic control EIS spectra, while the biotic EIS spectra required a two-time constant circuit. The fitted parameters are summarized in Table 2. The biotic impedance spectra for the three different headspace volumes (150 ml, 200 ml and 300 ml) fitted well with a two-time constant circuit model. The capacitors in the circuit model were not ideal capacitors. Thus, constant phase elements (CPE_s_) were used instead with n values in Table 2 indicating how close the CPEs (constant phase elements) were to capacitors (n = 1). The biotic equivalent circuit in Figure 6B contains: 1) solution resistance (*R*_s_), 2) a parallel combination of charge transfer resistance (*R*_ct_) and CPE_1_ (*Q*_dl_) associated with the metal surface electric double layer, 3) a parallel combination of biofilm resistance (*R*_f_) and CPE_2_ (*Q*_f_) associated with the biofilm/corrosion product layer on the X80 steel surface. The abiotic equivalent circuit in Figure 6A is simpler without *R*_f_ and *Q*_f_.

**TABLE 2:** Electrochemical parameters obtained from fitting EIS spectra in Figure 5.

Headspace (ml)	Day	*R*_s_ (Ω cm^2^)	*Q*_*f*_ (Ω^−1^ cm^−2^ s^n^)	*n*_*f*_	*R*_f_ (Ω cm^2^)	*Q*_*dl*_ (Ω^−1^ cm^−2^ s^n^)	*n*_*dl*_	*R*_ct_ (kΩ cm^2^)	$x^{2}$ (10^–3^)
Abiotic	1	11				4.26 × 10^–4^	0.82	17.7	3.10
	3	13				2.43 × 10^–4^	0.87	16.8	1.35
	7	13				4.07 × 10^–4^	0.87	16.2	4.06
	10	12				4.17 × 10^–3^	0.88	15.7	3.24
	14	13				3.81 × 10^–3^	0.88	15.3	2.91
150	1	24	1.41 × 10^–4^	0.89	4	1.01 × 10^–3^	0.71	6.78	0.61
	3	20	4.76 × 10^–3^	0.78	11	1.14 × 10^–3^	0.97	1.91	2.32
	7	18	5.11 × 10^–3^	0.74	12	3.24 × 10^–3^	0.87	5.76	3.38
	10	19	5.24 × 10^–3^	0.73	12	2.07 × 10^–3^	0.97	9.87	7.92
	14	24	4.03 × 10^–3^	0.78	7	6.13 × 10^–3^	0.88	9.98	5.31
200	1	47	1.01 × 10^–4^	0.88	41	1.47 × 10^–3^	0.80	5.88	4.85
	3	28	1.51 × 10^–3^	0.63	121	2.40 × 10^–3^	0.68	2.21	0.43
	7	34	7.62 × 10^–3^	0.72	180	1.13 × 10^–2^	0.71	1.32	5.29
	10	36	8.07 × 10^–3^	0.68	259	9.11 × 10^–3^	0.76	4.87	4.49
	14	37	7.04 × 10^–3^	0.64	129	2.93 × 10^–3^	0.75	5.63	2.47
300	1	47	1.83 × 10^–4^	0.85	39	1.85 × 10^–3^	0.84	5.63	0.39
	3	35	4.51 × 10^–3^	0.74	152	2.36 × 10^–3^	0.93	0.94	2.29
	7	37	3.80 × 10^–3^	0.75	115	6.38 × 10^–3^	0.92	0.86	1.89
	10	37	3.73 × 10^–3^	0.74	88	7.43 × 10^–3^	0.98	1.16	10.4
	14	38	3.31 × 10^–3^	0.76	17	1.41 × 10^–2^	0.80	0.96	8.17

Compared with charge resistance (*R*_ct_) values, the film resistance *R*_f_ values were quite small. However, the *R*_f_ values became larger with the increasing headspace volume due to *D. vulgaris* becoming more corrosive, which is consistent with the increased sessile cell count (Figure 2). *R*_ct_ was rate limiting in this study because it was much larger than *R*_s_ and *R*_f_. (*R*_ct_ + *R*_f_) is often used as the equivalent to *R*_p_ in qualitative corrosion analysis. In this work, (*R*_ct_ + *R*_f_) was the smallest for 300 ml headspace in Table 2, indicating the highest corrosion rate.

**FIGURE 7:**
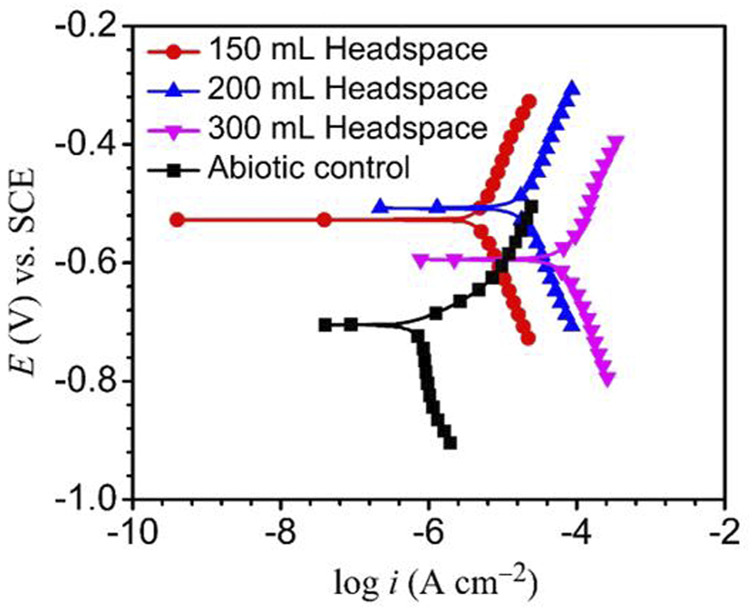
Potentiodynamic polarization curves at end of 14-day incubation with SRB, and without SRB (abiotic control).

The Tafel plots of X80 are shown in Figure 7. The corrosion current densities from the Tafel analysis of the potentiodynamic polarization curves are listed in Table 3. After the 14 days of SRB incubation, the coupon for the 300 ml headspace had the highest corrosion current density (*i*_corr_) of 74.8 μA cm^−2^ (Table 3), compared to 19.1 μA cm^−2^ (for 200 ml) and 4.8 μA cm^−2^ (for 150 ml). The abiotic *i*_corr_ in the deoxygenated ATCC 1249 culture medium was 0.79 μA cm^−2^, which was negligibly small. The corrosion current density trend here corroborates the *R*_p_^−1^ trend in Figure 4B and (*R*_ct_ + *R*_f_)^−1^ trend in Table 2. Thus, all the electrochemical corrosion data trends, with the exception of OCP, are consistent with the weight loss trend, all pointing to more sessile cells for faster MIC, which is characteristic of EET-MIC.

**TABLE 3:** Fitted electrochemical parameters from Tafel analysis at the end of the 14-day incubation in Figure 7.

Headspace (ml)	*i*_corr_ (μA cm^−2^)	*E*_corr_ (mV) vs. SCE	*β*_a_ (mV dec^−1^)	*β*_c_ (mV dec^−1^)
Abiotic	0.8	−739	125	−675
150	4.8	−550	314	−299
200	19.1	−520	328	−322
300	74.8	−590	387	−279

### H_2_S concentration and total gas pressure in headspace of anaerobic bottle with dogbone coupon

**FIGURE 8:**
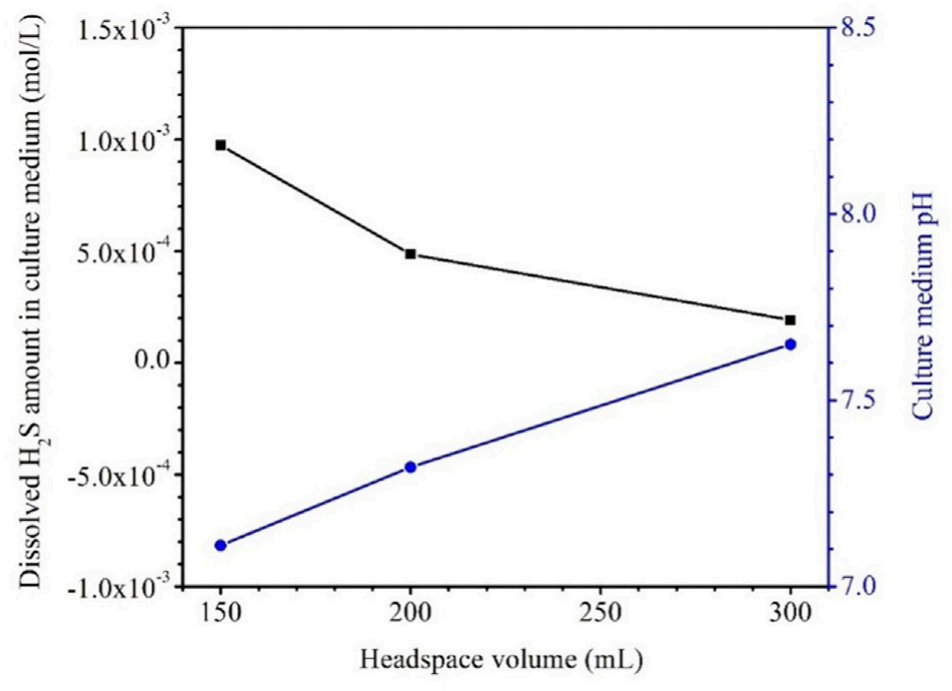
Dissolved [H_2_S] in both and broth pH after 14-day incubation in bottles with fixed 150 ml broth and varied headspace volume.

Table 4 shows that the H_2_S concentrations in the headspace gas phases for the anaerobic bottles (each containing one dogbone coupon) with headspace volumes of 150 ml, 200 ml and 300 ml were 8.50 × 10^3^ ppm (v/v), 7.75 × 10^3^ ppm, and 7.28 × 10^3^ ppm, respectively after the 14-day SRB incubation. The corresponding H_2_S concentration in the liquid phase was estimated based on H_2_S equilibrium at 37°C according to a published report (Ning et al., 2014). The dissolved [H_2_S] values for the headspace volumes of 150 ml, 200 ml and 300 ml were 1.06 mM, 0.95 mM and 0.84 mM, respectively (Figure 8; Table 4). As expected, a larger headspace allowed more H_2_S to escape from the liquid phase in order to reach a different H_2_S equilibrium between the gas and liquid phases. Figure 8 also shows that the final broth pH values were 7.08, 7.26, and 7.54 corresponding to headspace volumes of 150 ml, 200 ml and 300 ml, respectively. The broth pH increased slightly with the increasing headspace volume, because a larger headspace allowed more H_2_S to escape and this took away more protons from the broth as shown in Reaction (5). According to the following reaction (Jia et al., 2019a),

$${\text{HS}}^{-}{+H}^{+}⇌H_{2}S$$ (5)

**TABLE 4:** Data obtained and calculated for dogbone coupons with different headspace volumes (fixed 150 ml broth volume) after 14-day incubated in anaerobic bottles.

Headspace volume (mL)	H_2_S concentration in headspace (10^3^ ppm) (v/v)	Total pressure in headspace (bar)	H_2_S partial pressure in headspace (10^–2^ bar)	Dissolved [H_2_S] in liquid phase (10^–4^ M)
150	8.50	1.70	1.32	9.73
200	7.75	1.66	0.66	4.86
300	7.28	1.58	0.26	1.92

The dissolved [H_2_S] in the broth became lower with the increasing headspace volume while the pH value became higher (Table 4; Figure 8). All the pH values in this work were above and not far from 7. This is different from abiotic H_2_S corrosion studies, in which researchers introduce exogenous H_2_S to an aqueous solution and thus resulting in acidic pH, which is needed to cause appreciable abiotic H_2_S corrosion (Sun and Nesic, 2007).

### Sessile cell counts on dogbone coupons

**FIGURE 9:**
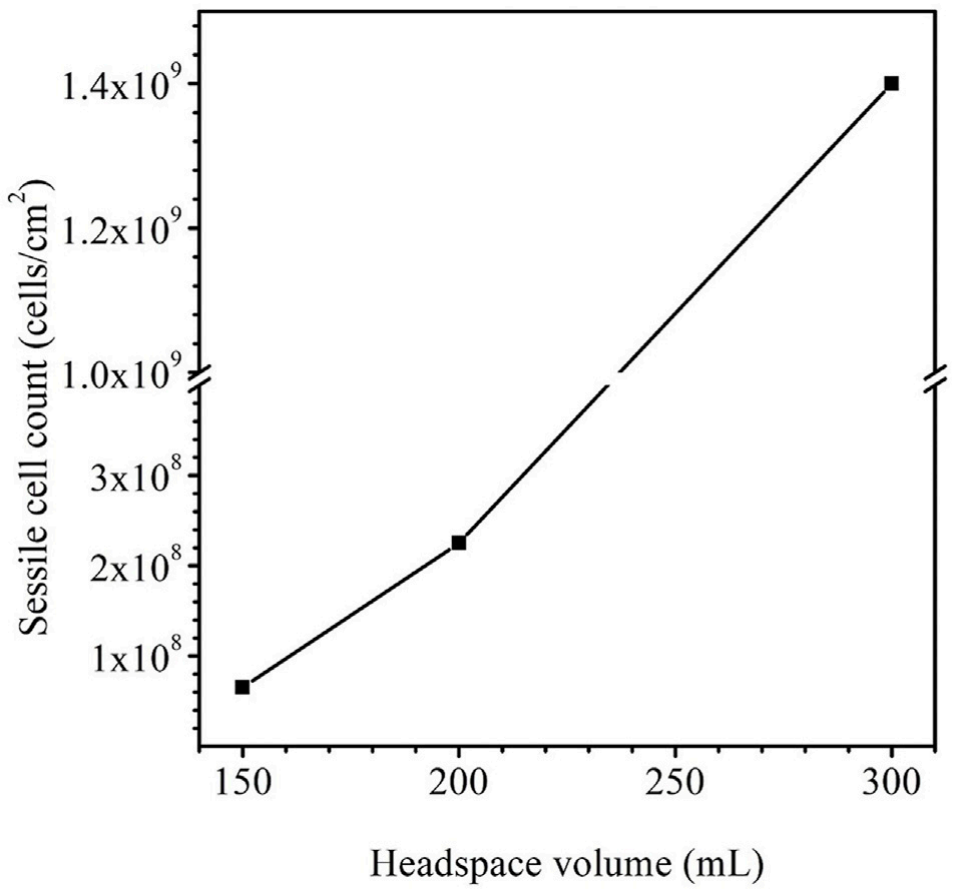
Sessile cell counts on dogbone coupons after 14-day incubation in anaerobic bottles with fixed 150 ml broth and varied headspace volume.

After the 14-day incubation, the sessile cell count was found to be higher in the anaerobic bottle with a larger headspace volume (Figure 9). The cell counts on coupons in the bottles with the headspace volumes of 150 ml, 200 ml and 300 ml were 6.5×10^7^ cells cm^−2^, 2.3×10^8^ cells cm^−2^ and 1.4×10^9^ cells cm^−2^, respectively. The increasing sessile cell count trend agrees with the decreasing dissolved [H_2_S] in Table 4. Decreased [H_2_S] means less toxicity and thus better sessile cell growth (Jia et al., 2019b). Although the 300 ml headspace bottle had lower H_2_S concentrations in both the gas and the liquid phases, its total amount (1.47 × 10^–4^ mol) was higher than in the bottles with 150 ml and 200 ml headspace volumes. This was reasonable because less H_2_S toxicity allowed better SRB growth and thus produced more H_2_S in the total amount in the liquid and headspace of a sealed anaerobic bottle.

### Pit depths on dogbone coupons

**FIGURE 10:**
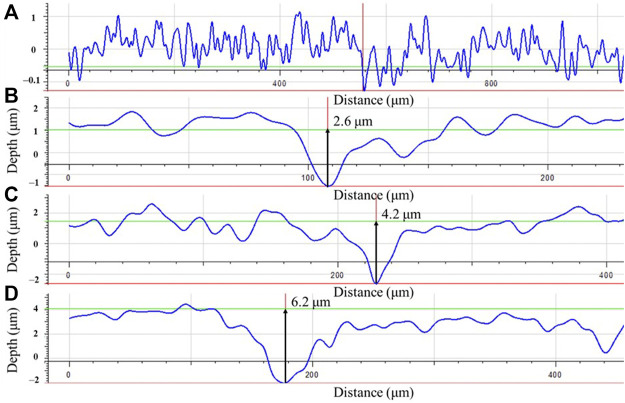
Maximum pit depths on dogbone coupons after 14-day incubation in bottles with headspace volumes of: **(A)** 150 ml (abiotic control), **(B)** 150 ml, **(C)** 200 ml, and **(D)** 300 ml, respectively.

Coupon surface morphologies on dogbone coupons after the 14-day incubation with biofilms and corrosion products removed were examined under IFM. Figure 10A shows that the abiotic coupon surface exhibited polished coupon surface roughness (y-scale enlarged to show details). For the biotic dogbone coupons, the maximum pit depth increased with a larger headspace volume in Figures 10B–D. They were 2.6 μm, 4.2 μm and 6.2 μm for headspace volumes of 150 ml, 200 ml and 300 ml, respectively. The pit depth trend here is consistent with the weight loss data trend. In future studies, pit density should be investigated as well (Javed et al., 2020a; Javed et al., 2020b). With a larger headspace, there was a lower amount of dissolved [H_2_S] and more sessile cells, which led to higher weight loss and deeper pits. The maximum pit depth increased by 58% when the headspace increased from 150 ml to 300 ml, while the broth volume was fixed at 150 ml.

### Tensile testing using dogbone coupons

**FIGURE 11:**
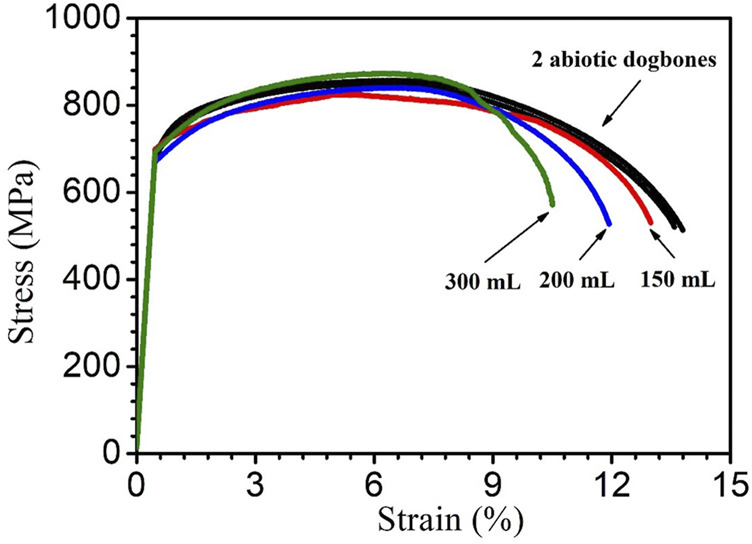
Stress–strain curves for 2 replicate abiotic X80 dogbone coupons and dogbone coupons (with corrosion products removed) obtained after 14-day incubation with SRB.

Figure 11 shows the stress-strain curves of X80 dogbone coupons. The dogbone coupons were retrieved after they had been immersed in SRB bottles with fixed 150 ml culture medium volume and varied headspace volumes (150 ml, 200 ml and 300 ml) for 14 days at 37°C. The ultimate (tensile) strength is the maximum stress that a material can withstand before final failure (Thamma and Jantasorn, 2022). It is the highest point of the *Y*-axis in Figure 11. The ultimate strain (elongation at break) demonstrates the ability of a material to resist shape change before finally breaking (Tian et al., 2021). It is the largest value of the *X*-axis (strain) in Figure 11 (Sluzalec, 1992). Lowering of these parameters can reflect the mechanical property degradation of the material under different conditions such as different MIC severity.

The ultimate strength of abiotic control X80 carbon steel was 853 ± 3 MPa. The ultimate tensile strength values of the abiotic dogbone coupon, and biotic dogbone coupons from bottles with different headspace volumes were all quite close as shown in Figure 11. Compared with the abiotic dogbone, in the presence of SRB with headspace volumes of 150 ml, 200 ml 300 ml, the ultimate strength losses were 3%, 2% and 0%, respectively (Table 5). These values were rather small. On the other hand, ultimate strain was reduced in the presence of SRB. Compared with the abiotic dogbone, in the presence of SRB with headspace volumes of 150 ml, 200 ml 300 ml, the ultimate strain losses were 6%, 13% and 23%, respectively (Table 5). With an increased headspace, MIC severity increased, making X80 steel more brittle. The corrosion damage by SRB pitting was the main factor in its mechanical property degradation study. H_2_S was unlike the driving force behind the relatively large ultimate strain loss, because in this work, more severe MIC corresponded with lower [H_2_S] in the broth.

**TABLE 5:** Ultimate tensile strength and ultimate tensile strain data from Figure 11.

Headspace volume (ml)	Ultimate tensile strength (MPa) (and loss)	Ultimate tensile strain (%) (and loss)
Abiotic dogbone	853 ± 3 (control)	13.7 ± 0.1% (control)
150	824 (3% loss)	12.9% (6% loss)
200	840 (2% loss)	11.9% (13% loss)
300	872 (0% loss)	10.5% (23% loss)

## Conclusion

(1) The tensile testing results show that the presence of SRB made the X80 steel more brittle which was reflected by the relatively large ultimate strain losses, compared to the abiotic control. Meanwhile, the ultimate strength loss was small (up to only 3%) for all the dogbone coupons after the 14-day incubation.
(2) More severe MIC weight loss and pitting led to more ultimate strain loss (up to 23%) in X80.
(3) This work confirms that in an anaerobic bottle with SRB, a larger headspace allows more H_2_S to escape from the broth, and this reduces the H_2_S toxicity in the broth and thus promoting sessile SRB growth. Increased sessile cell count leads to more severe weight loss and MIC pitting, which is consistent with EET-MIC.ASTM-E8/E8M-13a, 2013.

## Data Availability

The original contributions presented in the study are included in the article/supplementary material, further inquiries can be directed to the corresponding author.

## References

[B1] Alamri A. H. (2020). Localized corrosion and mitigation approach of steel materials used in oil and gas pipelines–An overview. Eng. Fail. Anal. 116, 104735. 10.1016/j.engfailanal.2020.104735

[B2] Astm-E8/E8M-13a (2013). Standard test methods for tension testing of metallic materials. West Conshohocken, PA: ASTM International.

[B3] Beavers J. A. Thompson N. G. (2006). External corrosion of oil and natural gas pipelines. ASM Handb. 13.

[B4] Cai Z. Xu J. Wei B. Sun C. (2022). A comparative study of sulfate-reducing *Desulfovibrio desulfuricans* induced corrosion behaviors in Q235, X65, X70, and X80 pipeline steels. Int. J. Press. Vessels Pip. 195, 104599. 10.1016/j.ijpvp.2021.104599

[B5] Cpuc and DOGGR (2019). California public utilities commission (CPUC) and department of conservation’s division of oil, gas, and geothermal resources (DOGGR). Available at: https://docs.cpuc.ca.gov/PublishedDocs/Published/G000/M292/K947/292947433.PDF.

[B6] Dou W. Liu J. Cai W. Wang D. Jia R. Chen S. (2019). Electrochemical investigation of increased carbon steel corrosion via extracellular electron transfer by a sulfate reducing bacterium under carbon source starvation. Corros. Sci. 150, 258–267. 10.1016/j.corsci.2019.02.005

[B7] Eaktasang N. Kang C. S. Lim H. Kwean O. S. Cho S. Kim Y. (2016). Production of electrically-conductive nanoscale filaments by sulfate-reducing bacteria in the microbial fuel cell. Bioresour. Technol. 210, 61–67. 10.1016/j.biortech.2015.12.090 26818576

[B8] Enning D. Garrelfs J. (2014). Corrosion of iron by sulfate-reducing bacteria: New views of an old problem. Appl. Environ. Microbiol. 80, 1226–1236. 10.1128/AEM.02848-13 24317078 PMC3911074

[B9] Fu Q. Xu J. Wei B. Qin Q. Bai Y. Yu C. (2022). Mechanistic diversity between nitrate and nitrite on biocorrosion of X80 pipeline steel caused by *Desulfovibrio desulfurican* and *Pseudomonas stutzeri* . Corros. Sci. 207, 110573. 10.1016/j.corsci.2022.110573

[B10] Gu T. Wang D. Lekbach Y. Xu D. (2021). Extracellular electron transfer in microbial biocorrosion. Curr. Opin. Electrochem. 29, 100763. 10.1016/j.coelec.2021.100763

[B11] Gu T. Xu D. Zhang P. Li Y. Lindenberger A. L. (2015). Microbiologically influenced corrosion and its impact on metals and other materials. Microbiol. Minerals, Metals, Mater. Environ. 25, 383–408. 10.1016/j.engfailanal.2020.104735

[B12] Huang L. Chang W. Zhang D. Huang Y. Li Z. Lou Y. (2022). Acceleration of corrosion of 304 stainless steel by outward extracellular electron transfer of *Pseudomonas aeruginosa* biofilm. Corros. Sci. 199, 110159. 10.1016/j.corsci.2022.110159

[B13] Jacobson G. A. (2007). Corrosion at prudhoe bay: A lesson on the line. Mater. Perform. 46.

[B14] Javaherdashti R. (2011). Impact of sulphate-reducing bacteria on the performance of engineering materials. Appl. Microbiol. Biotechnol. 91, 1507–1517. 10.1007/s00253-011-3455-4 21786108

[B15] Javed M. A. Neil W. C. McAdam G. Moreau J. W. Wade S. A. (2020a). Microbiologically influenced corrosion of stainless steel by sulfate reducing bacteria: A tale of caution. Corrosion 76, 639–653. 10.5006/3467

[B16] Javed M. A. Rieders N. Beech I. Avci R. Neil W. C. Wade S. A. (2020b). The influence of chemical cleaning methods on pitting morphology attributed to microbially influenced corrosion of stainless steels. Corrosion 77, 276–286. 10.5006/3707

[B17] Jia R. Tan J. L. Jin P. Blackwood D. J. Xu D. Gu T. (2018). Effects of biogenic H_2_S on the microbiologically influenced corrosion of C1018 carbon steel by sulfate reducing *Desulfovibrio vulgaris* biofilm. Corros. Sci. 130, 1–11. 10.1016/j.corsci.2017.10.023

[B18] Jia R. Wang D. Jin P. Unsal T. Yang D. Yang J. (2019a). Effects of ferrous ion concentration on microbiologically influenced corrosion of carbon steel by sulfate reducing bacterium *Desulfovibrio vulgaris* . Corros. Sci. 153, 127–137. 10.1016/j.corsci.2019.03.038

[B19] Jia R. Yang D. Abd Rahman H. B. Gu T. (2017). Laboratory testing of enhanced biocide mitigation of an oilfield biofilm and its microbiologically influenced corrosion of carbon steel in the presence of oilfield chemicals. Int. Biodeterior. Biodegrad. 125, 116–124. 10.1016/j.ibiod.2017.09.006

[B20] Jia R. Yang D. Dou W. Liu J. Zlotkin A. Kumseranee S. (2019b). A sea anemone-inspired small synthetic peptide at sub-ppm concentrations enhanced biofilm mitigation. Int. Biodeterior. Biodegrad. 139, 78–85. 10.1016/j.ibiod.2018.11.009

[B21] Li X. Lan S.-m. Zhu Z.-p. Zhang C. Zeng G.-m. Liu Y.-g. (2018a). The bioenergetics mechanisms and applications of sulfate-reducing bacteria in remediation of pollutants in drainage: A review. Ecotoxicol. Environ. Saf. 158, 162–170. 10.1016/j.ecoenv.2018.04.025 29684746

[B22] Li Y. Xu D. Chen C. Li X. Jia R. Zhang D. (2018b). Anaerobic microbiologically influenced corrosion mechanisms interpreted using bioenergetics and bioelectrochemistry: A review. J. Mater. Sci. Technol. 34, 1713–1718. 10.1016/j.jmst.2018.02.023

[B23] Li Z. Yang J. Guo H. Kumseranee S. Punpruk S. Mohamed M. E. (2022). Carbon source starvation of a sulfate-reducing bacterium–elevated MIC deterioration of tensile strength and strain of X80 pipeline steel. Adv. Mater. Toward Anti-Corrosion Anti-Biofoulings 8, 794051211. 10.3389/fmats.2021.794051

[B24] López-Celvera S. A. Orozco-Cruz R. Mejía-Sánchez E. Contreras-Cuevas A. Galván-Martínez R. (2018). Corrosion of API X80 steel immersed in seawater: Application of electrochemical noise technique. ECS Trans. 84, 117–124. 10.1149/08401.0117ecst

[B25] Lovley D. R. Phillips E. J. (1994). Novel processes for anaerobic sulfate production from elemental sulfur by sulfate-reducing bacteria. Appl. Environ. Microbiol. 60, 2394–2399. 10.1128/aem.60.7.2394-2399.1994 16349323 PMC201662

[B26] Lv M. Du M. (2018). A review: Microbiologically influenced corrosion and the effect of cathodic polarization on typical bacteria. Rev. Environ. Sci. Biotechnol. 17, 431–446. 10.1007/s11157-018-9473-2

[B27] Ning J. Zheng Y. Young D. Brown B. Nešić S. (2014). Thermodynamic study of hydrogen sulfide corrosion of mild steel. Corrosion 70, 375–389. 10.5006/0951

[B28] Peck H. D. (1993). Bioenergetic strategies of the sulfate-reducing bacteria, The sulfate-reducing bacteria: Contemporary perspectives. Manhattan, New York City: Springer. 10.1007/978-1-4613-9263-7_3

[B29] Pereira R. F. d. C. de Oliveira E. S. Lima M. A. G. d. A. Urtiga Filho S. L. (2021). Evaluation of the multi-structural potential of Ni-Co/SiC nanocomposite coatings electrodeposited in API 5L X80 steel. Mat. Res. 24. 10.1590/1980-5373-MR-2020-0362

[B30] Promnuan K. Sompong O. (2017). Biological hydrogen sulfide and sulfate removal from rubber smoked sheet wastewater for enhanced biogas production. Energy Procedia 138, 569–574. 10.1016/j.egypro.2017.10.161

[B31] Saad-Eldeen S. Garbatov Y. Soares C. G. (2012). Effect of corrosion degradation on ultimate strength of steel box girders. Corros. Eng. Sci. Technol. 47, 272–283. 10.1179/1743278212Y.0000000005

[B32] Salgar-Chaparro S. J. Darwin A. Kaksonen A. H. Machuca L. L. (2020). Carbon steel corrosion by bacteria from failed seal rings at an offshore facility. Sci. Rep. 10, 12287. 10.1038/s41598-020-69292-5 32703991 PMC7378185

[B33] Sheng J. Xia J. (2017). Effect of simulated pitting corrosion on the tensile properties of steel. Constr. Build. Mater. 131, 90–100. 10.1016/j.conbuildmat.2016.11.037

[B34] Sherman S. Brownlee D. Kakadjian S. (2015). “MIC related coil tubing failures and equipment damage,” in Proceedings of the SPE/ICoTA Coiled Tubing & Well Intervention Conference & Exhibition, Woodlands, Texas, USA. 10.2118/173658-MS

[B35] Sluzalec A. (1992). Introduction to nonlinear thermomechanics. NASA STI/Recon Tech. Rep. A 92, 48425. 10.1007/978-1-4471-1906-7

[B36] Smith N. W. Shorten P. R. Altermann E. Roy N. C. McNabb W. C. (2019). A mathematical model for the hydrogenotrophic metabolism of sulphate-reducing bacteria. Front. Microbiol. 10, 1652. 10.3389/fmicb.2019.01652 31379794 PMC6653664

[B37] Sun W. Nesic S. (2007). *A mechanistic model of H* _2_ *S corrosion of mild steel* . CORROSION 2007, Paper No. 07655. NACE International.

[B38] Tang F. Lin Z. Chen G. Yi W. (2014). Three-dimensional corrosion pit measurement and statistical mechanical degradation analysis of deformed steel bars subjected to accelerated corrosion. Constr. Build. Mater. 70, 104–117. 10.1016/j.conbuildmat.2014.08.001

[B39] Thamma U. Jantasorn P. (2022). Effects of shear deformation via torsion on tensile strain-hardening behavior of SCM415 low-alloy steel. Mater. Today Proc. 52, 2496–2500. 10.1016/j.matpr.2021.10.437

[B40] Thauer R. K. Stackebrandt E. Hamilton W. A. (2007). Energy metabolism and phylogenetic diversity of sulphate-reducing bacteria. Sulphate-reducing Bact. 1, 1–38. 10.1017/CBO9780511541490

[B41] Tian F. Pan L. (2021). Effect of glutaraldehyde on corrosion of X80 pipeline steel. Coatings 11, 1176. 10.3390/coatings11101176

[B42] Tian J. Li C. Xian G. (2021). Reciprocating friction and wear performances of nanometer sized‐TiO_2_ filled epoxy composites. Polym. Compos. 42, 2061–2072. 10.1002/pc.25959

[B43] Tran Thi Thuy T. Kannoorpatti K. Padovan A. Thennadil S. (2020). Effect of alkaline artificial seawater environment on the corrosion behaviour of duplex stainless steel 2205. Appl. Sci. 10, 5043. 10.3390/app10155043 PMC7890476 33614061

[B44] Unsal T. Ilhan-Sungur E. Arkan S. Cansever N. (2016). Effects of Ag and Cu ions on the microbial corrosion of 316L stainless steel in the presence of *Desulfovibrio* sp. Bioelectrochemistry 110, 91–99. 10.1016/j.bioelechem.2016.03.008 27105168

[B45] Unsal T. Wang D. Kijkla P. Kumseranee S. Punpruk S. Mohamed M. E. (2022). Food-grade D-limonene enhanced a green biocide in the mitigation of carbon steel biocorrosion by a mixed-culture biofilm consortium. Bioprocess Biosyst. Eng. 45, 669–678. 10.1007/s00449-021-02685-6 34997847

[B46] Vaidya R. Butt D. Hersman L. Zurek A. (1997). Effect of microbiologically influenced corrosion on the tensile stress-strain response of aluminum and alumina-particle reinforced aluminum composite. Corrosion 53, 136–141. 10.5006/1.3280447

[B47] Wang D. Ivanova S. A. Hahn R. Gu T. (2022). Evaluation of trehalase as an enhancer for a green biocide in the mitigation of *Desulfovibrio vulgaris* biocorrosion of carbon steel. Bioprocess Biosyst. Eng. 45, 659–667. 10.1007/s00449-021-02684-7 34982209

[B48] Wang D. Liu J. Jia R. Dou W. Kumseranee S. Punpruk S. (2020). Distinguishing two different microbiologically influenced corrosion (MIC) mechanisms using an electron mediator and hydrogen evolution detection. Corros. Sci. 177, 108993. 10.1016/j.corsci.2020.108993

[B49] Wasim M. Djukic M. B. (2022104467). External corrosion of oil and gas pipelines: A review of failure mechanisms and predictive preventions. J. Nat. Gas Sci. Eng. 100, 104467. 10.1016/j.jngse.2022.104467

[B50] Wolodko J. Haile T. Khan F. Taylor C. Eckert R. Hashemi S. J. (2018). Modeling of microbiologically influenced corrosion (MIC) in the oil and gas industry-past, present and future. CORROSION 2018, Paper No. 11398. Phoenix, AZ: NACE International

[B51] Xu D. Gu T. (2014). Carbon source starvation triggered more aggressive corrosion against carbon steel by the *Desulfovibrio vulgaris* biofilm. Int. Biodeterior. Biodegrad. 91, 74–81. 10.1016/j.ibiod.2014.03.014

[B52] Xu D. Xia J. Zhou E. Zhang D. Li H. Yang C. (2017). Accelerated corrosion of 2205 duplex stainless steel caused by marine aerobic *Pseudomonas aeruginosa* biofilm. Bioelectrochemistry 113, 1–8. 10.1016/j.bioelechem.2016.08.001 27578208

[B53] Yousaf M. Ali I. Arif M. Mustafa G. Ahmad S. Afzal N. (2015). Effects of microbiologically influenced corrosion by Bacillus Megaterium bacteria on the mechanical properties of Al-Cu Alloy. Mater. Today Proc. 2, 5669–5673. 10.1016/j.matpr.2015.11.107

[B54] Zhang W. Li H.-J. Wang M. Wang L.-J. Pan Q. Ji X. (2019). Tetrahydroacridines as corrosion inhibitor for X80 steel corrosion in simulated acidic oilfield water. J. Mol. Liq. 293, 111478. 10.1016/j.molliq.2019.111478

[B55] Zhang Z. Xu S. Li R. (2020). Comparative investigation of the effect of corrosion on the mechanical properties of different parts of thin-walled steel. Thin-Walled Struct. 146, 106450. 10.1016/j.tws.2019.106450

[B56] Zhou E. Lekbach Y. Gu T. Xu D. (2022). Bioenergetics and extracellular electron transfer in microbial fuel cells and microbial corrosion. Curr. Opin. Electrochem. 31, 100830. 10.1016/j.coelec.2021.100830

